# A single transcript for the prognosis of disease severity in COVID-19 patients

**DOI:** 10.1038/s41598-021-91754-7

**Published:** 2021-06-09

**Authors:** Hongxing Lei

**Affiliations:** 1 CAS Key Laboratory of Genome Sciences and Information, Beijing Institute of Genomics, Chinese Academy of Sciences, and China National Center for Bioinformation, Beijing, China; 2 Cunji Medical School, University of Chinese Academy of Sciences, Beijing, China; 3 Center of Alzheimer’s Disease, Beijing Institute for Brain Disorders, Beijing, China

**Keywords:** Prognostic markers, Infectious diseases

## Abstract

With many countries strapped for medical resources due to the COVID-19 pandemic, it is highly desirable to allocate the precious resources to those who need them the most. Several markers have been found to be associated with the disease severity in COVID-19 patients. However, the established markers only display modest prognostic power individually and better markers are urgently needed. The aim of this study is to investigate the potential of *S100A12*, a prominent marker gene for bacterial infection, in the prognosis of disease severity in COVID-19 patients. To ensure the robustness of the association, a total of 1695 samples from 14 independent transcriptome datasets on sepsis, influenza infection and COVID-19 infection were examined. First, it was demonstrated that *S100A12* was a marker for sepsis and severity of sepsis. Then, *S100A12* was found to be a marker for severe influenza infection, and there was an upward trend of *S100A12* expression as the severity level of influenza infection increased. As for COVID-19 infection, it was found that *S100A12* expression was elevated in patients with severe and critical COVID-19 infection. More importantly, *S100A12* expression at hospital admission was robustly correlated with future quantitative indexes of disease severity and outcome in COVID-19 patients, superior to established prognostic markers including CRP, PCT, d-dimer, ferritin, LDH and fibrinogen. Thus, *S100A12* is a valuable novel prognostic marker for COVID-19 severity and deserves more attention.

## Introduction

The COVID-19 pandemic has caused major destruction to the entire world. There have been over 160 million cases and 3 million deaths reported so far (https://www.who.int/emergencies/diseases/novel-coronavirus-2019). Although the vast majority of COVID-19 patients have only mild or even no symptoms and require little medical attention, the sheer volume of hospitalized patients have put an unprecedented stress on the medical systems worldwide. To improve the survival rate, it is urgently needed to have better stratification of the patients admitted to the hospitals based on promising biomarkers.

Blood-derived prognostic biomarkers for COVID-19 infection have been heavily investigated. Inflammatory and immune factors were among the most widely studied, including serum IL-6 and TNF-α^1^, IFN-α^2^, d-dimer^3^, S100A8/A9 and HMGB1^4^, TNFR1 and TNFR2^5^, acetylated K676 TGFBIp^6^, progranulin (GRN)^7^, and sphingosine-1-phosphate^8^. Other factors include serum GDF-15^9^, calcium^10^, and fasting blood glucose^11^. Markers for myocardial injury^12^, endothelial cell and platelet activation^13,14^ have also been proposed. Higher antibody production has also been observed in severe COVID-19 patients^15^. Dynamic pattern of IL-6, C-reactive protein (CRP), fibrinogen, lactate dehydrogenase (LDH), platelet count and CD45 count may also be informative^16–18^.

Immune cell profiling uncovered the prognostic value of T cell subset counts^19^, neutrophil to lymphocyte ratio^20,21^ and immature neutrophil to VD2 T cell ratio^22^. Other observations include aberrant activation and dysregulation of CD8 + T cells^23^, CoV-2-specific CD4 + T helper cell^24^, and higher level of adaptive natural killer (NK) cells^25^. Additional factors include red blood cell distribution width^26^, PD-L1 expression in basophils and eosinophils^27^, and quantitation of plasma SARS-CoV-2 RNA^28–30^.

Since individual factors only displayed modest prognostic power, some groups attempted to derive composite models based on factors such as age, sex, lymphocyte counts, neutrophil counts, CRP, and procalcitonin (PCT)^31,32^. Others applied proteomics, metabolomics, and lipidomics to construct predictive panels of serum proteins, metabolites and lipids^33–36^. However, these models are difficult to interpret and have rarely been validated by independent groups.

Since none of the currently proposed prognostic markers have satisfactory performance, it is highly desirable to discover novel factors with better prognostic power. Certain factors may be unique to COVID-19 infection, but some factors are likely universal to all kinds of severe infection. Sepsis is a severe form of infection. Therefore, it is conceivable that certain prognostic markers for sepsis may be transferable to COVID-19 infection. In fact, viral sepsis was proposed as a mechanism for severe COVID-19 infection^37,38^. It has also been found that the most severe cases (including death) of COVID-19 infection indeed all had sepsis^39^. Among COVID-19 patients, septic patients had significantly abnormal immune profile including higher serum IL-6^40^. Many features were similar between bacterial sepsis and SARS-CoV-2 sepsis, although cytokine storm was generally milder in the latter case^41^. In addition, COVID-19 infection is a type of respiratory viral infection. So certain prognostic markers for other types of respiratory viral infection such as influenza infection may also be transferable to COVID-19 infection. The rationale of using datasets encompassing sepsis, influenza infection and COVID-19 infection is that common factors may exist in different types of infection and certain common factors may be transferable from one type of infection to another.

In our previous works on host response to infection, we proposed several genes as the signature for bacterial infection, among which *S100A12* was the most prominent marker^42–44^. To investigate the potential prognostic power of *S100A12* in COVID-19 infection, a three-step approach was applied. First, using RNA-Seq data from three independent studies on sepsis, I demonstrated that *S100A12* was a marker for sepsis and severity of sepsis. Then, using microarray data from six independent studies on influenza infection, I demonstrated that *S100A12* was a marker for severity of influenza infection. Finally, using RNA-Seq data from five independent studies on COVID-19 infection, I demonstrated that *S100A12* was indeed a valuable prognostic marker for COVID-19 severity.

## Materials and methods

### RNA-Seq datasets for sepsis

All three datasets were downloaded from NCBI gene expression omnibus (GEO, https://www.ncbi.nlm.nih.gov/geo/). All three datasets were derived from whole blood. Dataset **GSE154918** contains 105 samples, including 40 samples from healthy controls, 12 samples from patients with uncomplicated infection, 39 samples from sepsis patients, and 14 samples from follow-up of sepsis (no reference available yet). Dataset **GSE63042** contains 129 samples, including 23 samples from patients with systemic inflammatory response syndrome (SIRS), 24 samples from patients with uncomplicated sepsis (no disease progression), 21 samples from patients with severe sepsis (severe status at day 0 through day 3), 33 samples from patients with septic shock, and 28 samples from patients with sepsis death^45^. Sepsis is essentially SIRS plus infection. In addition, sepsis is highly heterogeneous due to the pathogen types, site of infection and many other factors. Dataset **GSE131411** contains 96 samples from 21 septic shock patients and 11 cardiogenic shock patients, where each patient was sampled at three time points^46^. Septic shock is caused by overwhelming systemic inflammation, while cardiogenic shock is caused by heart problem. In total, 328 samples were included in these three RNA-Seq studies on sepsis.

### Microarray datasets for respiratory viral infection

Six datasets were downloaded from GEO. All six datasets were derived from whole blood. Dataset **GSE27131** contains 21 samples, including 7 samples from healthy controls, 7 samples from patients with severe H1N1 infection at day zero, and 7 samples from patients with severe H1N1 infection at day six^47^. The severe H1N1 infection was defined as having bilateral chest X-ray infiltrates and requiring mechanical ventilators. Dataset **GSE21802** contains 40 samples, including 4 samples from healthy controls, 6 samples from patients with mechanical ventilation at the early course of severe H1N1 infection, 6 samples from patients without mechanical ventilation at the early course, 14 samples from patients with mechanical ventilation at the late course, and 10 samples from patients without mechanical ventilation at the late course^48^. All of the H1N1 patients were ICU patients with acute respiratory stress. Dataset **GSE40012** contains 150 samples, including 36 samples from healthy controls at day one and day five, 61 samples from patients with severe bacterial pneumonia at day one through day five, 39 samples from patients with severe H1N1 pneumonia at day one through day five, and 14 samples from patients with severe pneumonia caused by mixed bacterial and viral infection at day one through day five^49^. All of the pneumonia patients were ICU patients.

Dataset **GSE68310** contains 488 samples from patients with mild influenza infection in a community monitoring study where people were sampled at baseline, day zero through day twenty one of the symptom onset, and the next spring^50^. These individuals had influenza-like illness but did not have severe respiratory disease. Dataset **GSE101702** contains 159 samples, including 52 samples from healthy controls, 63 samples from patients with moderate influenza infection, and 44 samples from patients with severe influenza infection^51^. All of the patients had influenza-like illness. Case assignment was done retrospectively. Moderate infection was defined as having emergency department visit but requiring no invasive respiratory support. Severe infection was defined as having significant respiratory failure requiring mechanical ventilation. Dataset **GSE111368** contains 200 samples, including 130 samples from healthy controls, 29 samples from patients with H1N1 infection at tier one severity, 22 samples from patients with H1N1 infection at tier two severity, and 19 samples from patients with H1N1 infection at tier three severity^52^. All of the patients had influenza-like illness. Tier 1 severity was defined as having no substantial respiratory compromise and > 93% blood oxygen saturation. Tier 2 was defined as having < 93% blood oxygen saturation and requiring non-invasive oxygen support. Tier 3 was defined as respiratory compromise requiring invasive mechanical ventilation. In total, 1058 samples were included in these six datasets on influenza infection.

### RNA-Seq datasets for COVID-19

Three datasets were downloaded from GEO. Dataset **GSE152641** contains 86 samples derived from whole blood, including 24 samples from healthy controls and 62 samples from patients with COVID-19^53^. Dataset **GSE161731** contains 47 independent COVID-19 samples derived from whole blood, including 12 samples from hospitalized patients and 35 samples from non-hospitalized patients^54^. Dataset **GSE152418** contains 34 samples derived from peripheral blood mononuclear cells (PBMC), including 17 samples from healthy controls, 4 samples from patients with moderate COVID-19, 8 samples from patients with severe COVID-19, 4 samples from ICU patients with COVID-19, and one sample from a convalescent patient with COVID-19^55^. Dataset **GSE157103** contains 126 samples derived from leukocyte, including 100 samples from patients with COVID-19 and 26 samples from patients with other diseases^56^. All of the patients had symptoms compatible with COVID-19 infection. Additionally, another RNA-Seq study contains 16 samples derived from leukocytes, including 4 samples from healthy controls, 6 samples from patients with moderate COVID-19, and 6 samples from patients with severe COVID-19 (data at individual level not available yet)^57^. The disease severity in this study was based on WHO guideline. In total, 309 samples were included in these four RNA-Seq studies on COVID-19.

### Data analysis

The expression values of *S100A12* were extracted from the processed GEO data. For RNA-Seq data, the normalized counts were log transformed. For microarray data, the normalized expression values were also log transformed when necessary. The group comparison for each dataset was done in R (https://www.r-project.org/) using t-test. The figures were also drawn with R.

We shall note that the quality control of the data is intrinsic to the original study design (please refer to the original peer-reviewed publications for more details). For example, in the COVID-19 dataset GSE157103, only adult patients 18 years or older were enrolled, all of which had symptoms compatible with COVID-19 infection. Patients with imminent death were excluded. In addition, there were equal number of patients in the ICU group and non-ICU group, the mean age, mean BMI and proportion of male patients were similar in both groups, and so on. Blood collection and RNA-Seq experiment and data processing followed standard protocols, and two samples were excluded due to poor sequencing quality. In addition, we have found that *S100A12* expression is not affected by age or sex in our previous data analysis. The *S100A12* expression is also not significantly altered in non-infectious illness in our previous work.

In the original publication of the dataset GSE157103, the authors chose hospital free days at day 45 of hospital admission (HFD-45) as the disease severity index for COVID-19 infection for the following reasons, (1) being a single metric, (2) universally applicable to patients with different severity, (3) more suitable for COVID-19 infection, (4) compatible with COVID-19 development. Death was assigned as 0 HFD-45 value to reflect the disease severity.

## Results

### *S100A12* expression is an indicator of severe infection

**Figure 1:**
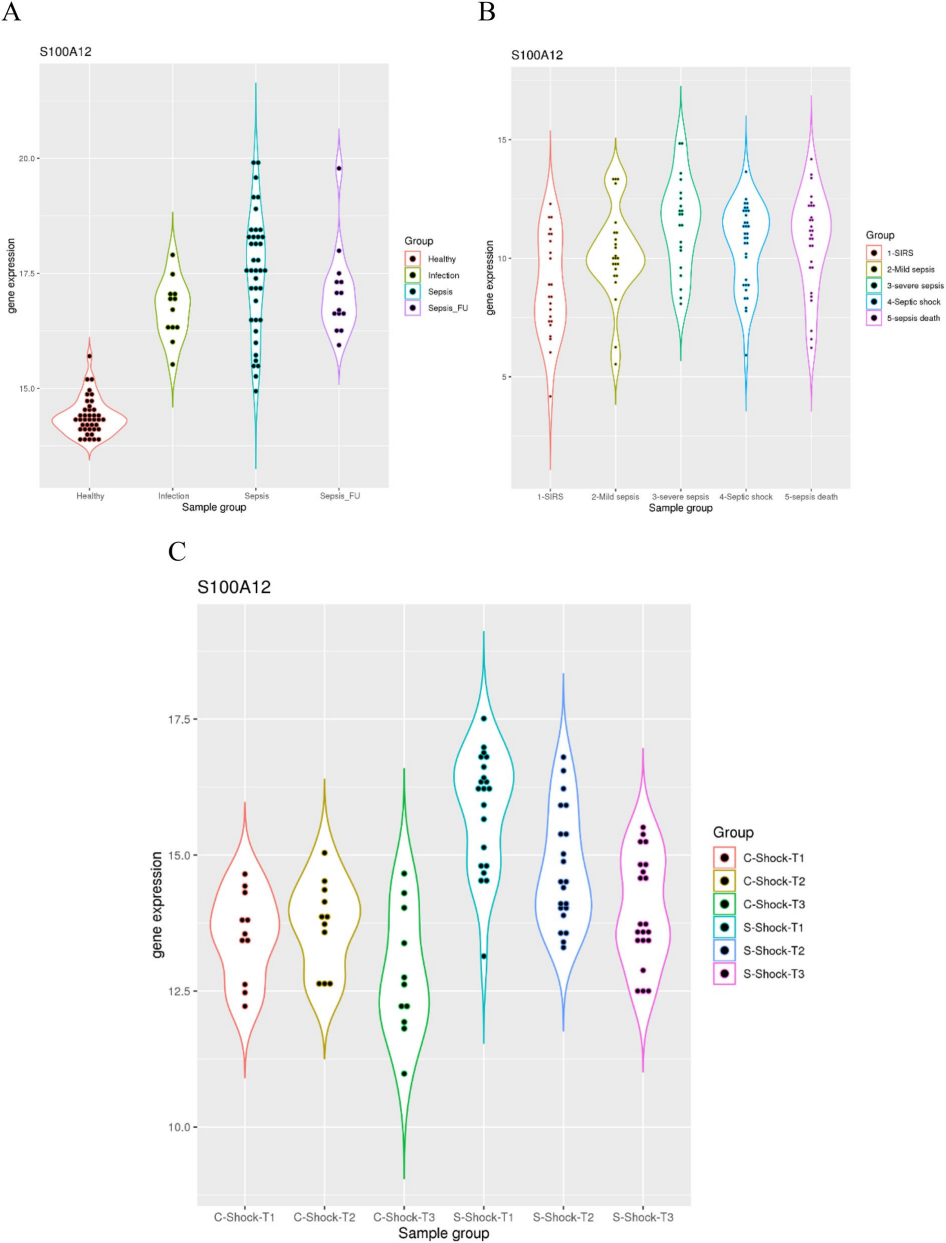
S100A12 as a marker for the severity of infection. (**A**) upper left, comparison of *S100A12* expression in healthy controls, patients with uncomplicated infection, patients with sepsis, and follow-up of sepsis patients. (**B**) upper right, comparison of *S100A12* expression in patients with SIRS, mild sepsis, severe sepsis, septic shock and sepsis death. (**C**) lower panel, comparison of *S100A12* expression in patients with cardiogenic shock and septic shock at three time points.

First, I examined whether *S100A12* expression can be a marker for severe infection especially sepsis. The dataset GSE654918 included samples from healthy controls, uncomplicated infection, sepsis and sepsis follow-up (Fig. 1A). It was evident that *S100A12* expression was significantly elevated in the uncomplicated infection group compared to healthy controls (*p* = 1.95e-8), where 11 of the 12 patients had higher *S100A12* expression than the highest value in the control group. The *S100A12* expression was further elevated in the sepsis group compared to the uncomplicated infection group (*p* = 0.0074), where 16 of the 39 sepsis patients (41%) had higher *S100A12* expression than the highest value in the uncomplicated infection group. In addition, *S100A12* expression was not significantly different between the sepsis follow-up group and the uncomplicated infection group (*p* = 0.26). Thus, *S100A12* expression is a clear indicator of severe infection.

The next question is whether *S100A12* expression is correlated with sepsis severity. The dataset GSE63042 included samples from SIRS, mild sepsis, severe sepsis, septic shock and sepsis death (Fig. 1B). It was evident that *S100A12* expression was significantly elevated in the mild sepsis group compared to the SIRS group (*p* = 0.027), further validating *S100A12* as a marker for infection. The *S100A12* expression was further elevated in the severe sepsis group compared to the mild sepsis group (*p* = 0.042). Additionally, no significant difference was found between severe sepsis and septic shock or septic death (*p* > 0.05 for both comparison). Therefore, *S100A12* expression is an indicator of severity in sepsis.

As a marker of disease severity, the dynamic pattern is also worth of investigation. The dataset GSE131411 included samples from three time points of septic shock and cardiogenic shock (Fig. 1C). It was evident that *S100A12* expression was significantly elevated in the septic group compared to the cardiogenic shock group at the first time point (*p* = 2.99e-7), again validating *S100A12* as a marker for infection. Interestingly, the *S100A12* expression was significantly decreased from T1 to T2 (*p* = 0.002) and also from T2 to T3 (*p* = 0.023) for patients with septic shock. In fact, 19 of the 21 patients with septic shock had this downward trend during the treatment process, while the other two patients had the lowest expression at the first time point (probably within the normal range). Thus, *S100A12* expression is an indicator of treatment progress in sepsis.

### *S100A12* expression is an indicator of severe influenza infection

**Figure 2:**
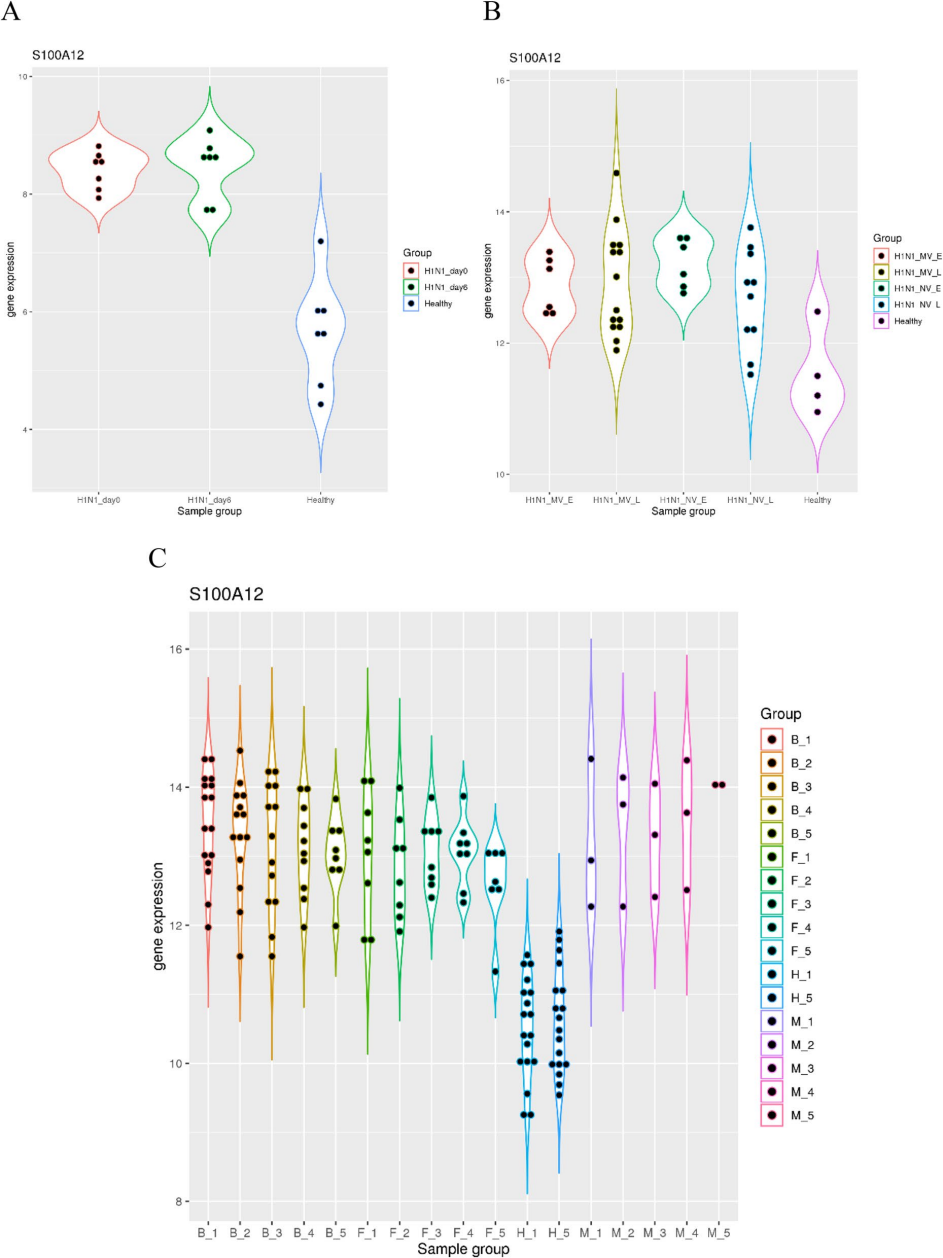
S100A12 as a marker for the severe influenza infection. (**A**) upper left, comparison of *S100A12* expression in healthy controls and patients with severe H1N1 infection at two time points. (**B**) upper right, comparison of *S100A12* expression in healthy controls and patients with severe H1N1 infection at two time points (early and late course of the infection). Patients with severe H1N1 infection were further divided into two groups (with or without mechanical ventilation). (**C**) lower panel, comparison of *S100A12* expression in healthy controls and patients with severe bacterial pneumonia, severe influenza pneumonia and severe pneumonia with mixed infection at five time points.

Next, I examined whether *S100A12* expression can be a marker for severe respiratory viral infection especially influenza infection. The dataset GSE27131 included samples from healthy controls and patients with severe H1N1 infection (defined as requiring mechanical ventilation) at day 0 and day 6 of ICU admission (Fig. 2A). It was evident that *S100A12* expression was significantly elevated in the H1N1 group compared to the control group at both time point (*p* = 9.85e-5 and 4.50e-5). In addition, all of the samples in the patient group had higher *S100A12* expression than the highest value in the control group.

Similar trend was observed in the dataset GSE21802 which also included samples from healthy controls and patients with severe H1N1 infection (Fig. 2B). Compared to the control group, patients with mechanical ventilation had significantly elevated *S100A12* expression at both time points (*p* = 0.019 and 0.014), and even patients without mechanical ventilation also had significantly elevated *S100A12* expression at both time points (*p* = 0.0086 and 0.031).

The elevation of *S100A12* expression in severe influenza infection can be compared to that in severe bacterial infection. The dataset GSE40012 included samples from healthy controls and patients with severe community-acquired pneumonia at day 0 through day 5 of ICU admission (Fig. 2C). Compared to the control group at both time points, the patients with severe bacterial pneumonia had significantly elevated *S100A12* expression at day one (*p* = 5.74e-13 and 1.79e-12). All of the samples in this group had higher *S100A12* expression than the highest value in the control groups, again validating *S100A12* expression as a prominent marker for bacterial infection. During the next four days, *S100A12* expression stayed high (*p* < 4.15e-8 for all the comparison against the control group). Compared to the control group at both time points, the patients with severe H1N1 pneumonia also had significantly elevated *S100A12* expression at day one (*p* = 2.58e-5 and 3.62e-5). During the next four days, *S100A12* expression also stayed high (*p* < 1.10e-5 for all the comparison against the control group). In addition, the patient group with mixed bacterial and viral infection also had significantly elevated *S100A12* expression compared to the control group (*p* < 0.046 for all the comparison). Thus, the three studies described above demonstrated that *S100A12* expression is a marker for severe influenza infection.

### Elevation of *S100A12* expression and the severity of influenza infection

**Figure 3:**
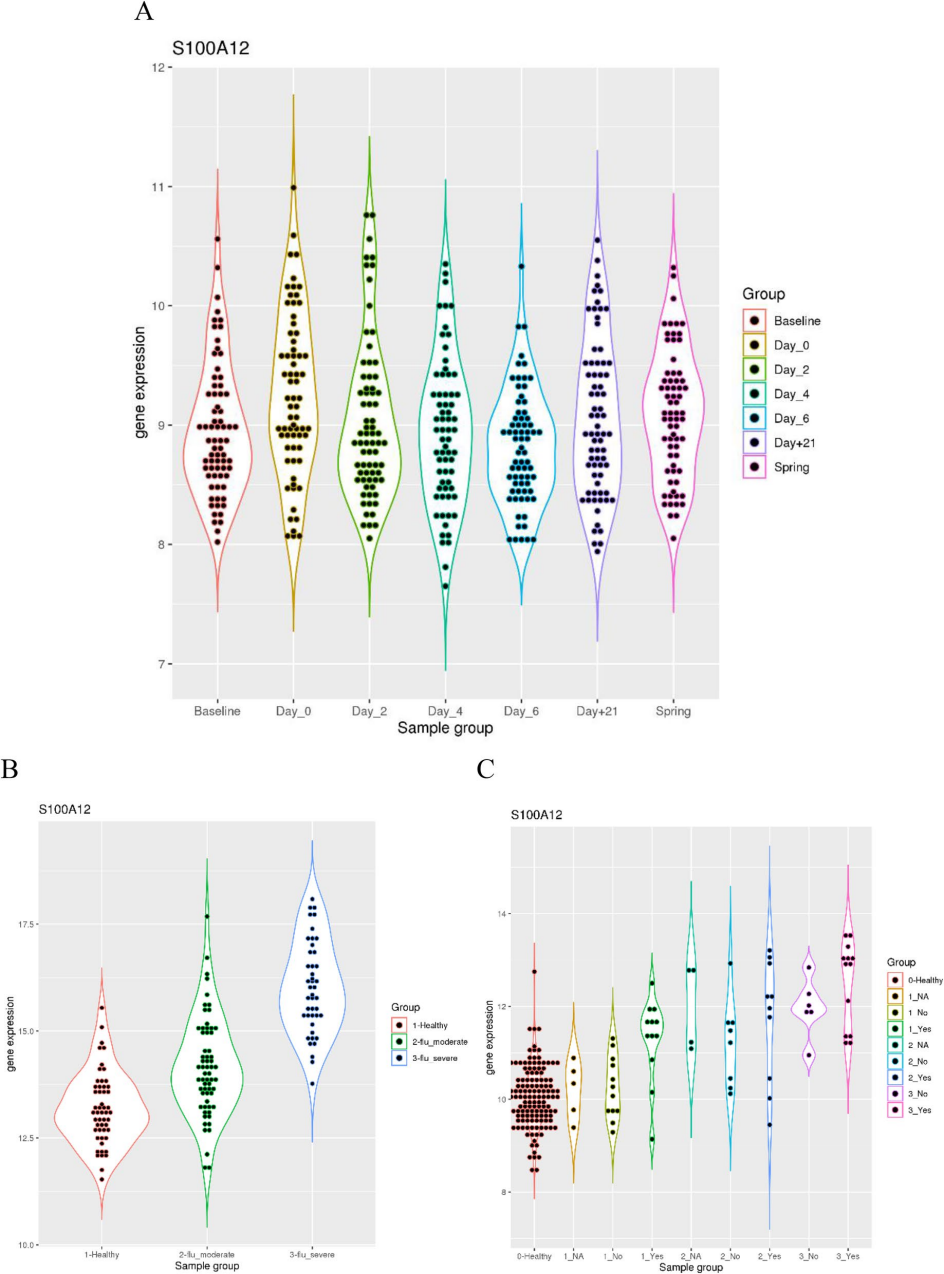
S100A12 as an indicator of the severity in influenza infection. (**A**) upper panel, comparison of *S100A12* expression in patients with mild influenza infection throughout a whole year of community monitoring (prior to and after the infection onset, and follow-up in the next spring). (**B**) lower left, comparison of *S100A12* expression in healthy controls and patients with moderate or severe influenza infection. (**C**) lower right, comparison of *S100A12* expression in healthy controls and patients with influenza infection at three severity levels. Some of the patients have known status of bacterial infection (Yes or No) while others don’t (NA).

Then, I further examined how *S100A12* expression is elevated at different severity levels of influenza infection. The dataset GSE68310 included samples from a prospective study of community monitoring, likely all with mild influenza infection. Although small fluctuation was observed in the *S100A12* expression, it stayed relatively constant throughout the whole year (*p* = 0.11 between day 0 of influenza infection and the next spring) (Fig. 3A). This is consistent with previous findings where the marker genes for respiratory viral infection are mainly interferon-stimulated genes (ISGs) such as *IFI27* and *RSAD2*. Thus, *S100A12* expression is not elevated in mild influenza infection.

As the severity level of influenza infection increases, we can observe higher level of *S100A12* expression. The dataset GSE101702 included samples from healthy controls and patients with influenza infection. Patients with moderate flu had significantly elevated *S100A12* expression compared to the control group (*p* = 1.03e-6) (Fig. 3B). In addition, patients with severe flu also had significantly elevated *S100A12* expression compared to the moderate flu group (*p* = 1.91e-13). This demonstrated a step-wise increase of *S100A12* expression as the severity level of influenza infection increased.

This trend was further supported by the dataset GSE111368 which included samples from healthy controls and patients with various severity levels of H1N1 infection, some of which had known status of bacterial co-infection. For patients at tier 1 severity level, they did not have significantly different *S100A12* expression compared to the controls unless they had confirmed bacterial co-infection (Fig. 3C). In contrast, patients at tier 2 and tier 3 levels had significantly elevated *S100A12* expression compared to the controls (*p* = 0.025, 0.0093 and 0.0029 for the patient groups at tier 2 level, and *p* = 0.00043 and 2.93e-7 for the patient groups at tier 3 level). This also validated the upward trend of *S100A12* expression as the severity of influenza infection increases.

### *S100A12* expression is an indicator of severe COVID-19 infection

**Figure 4:**
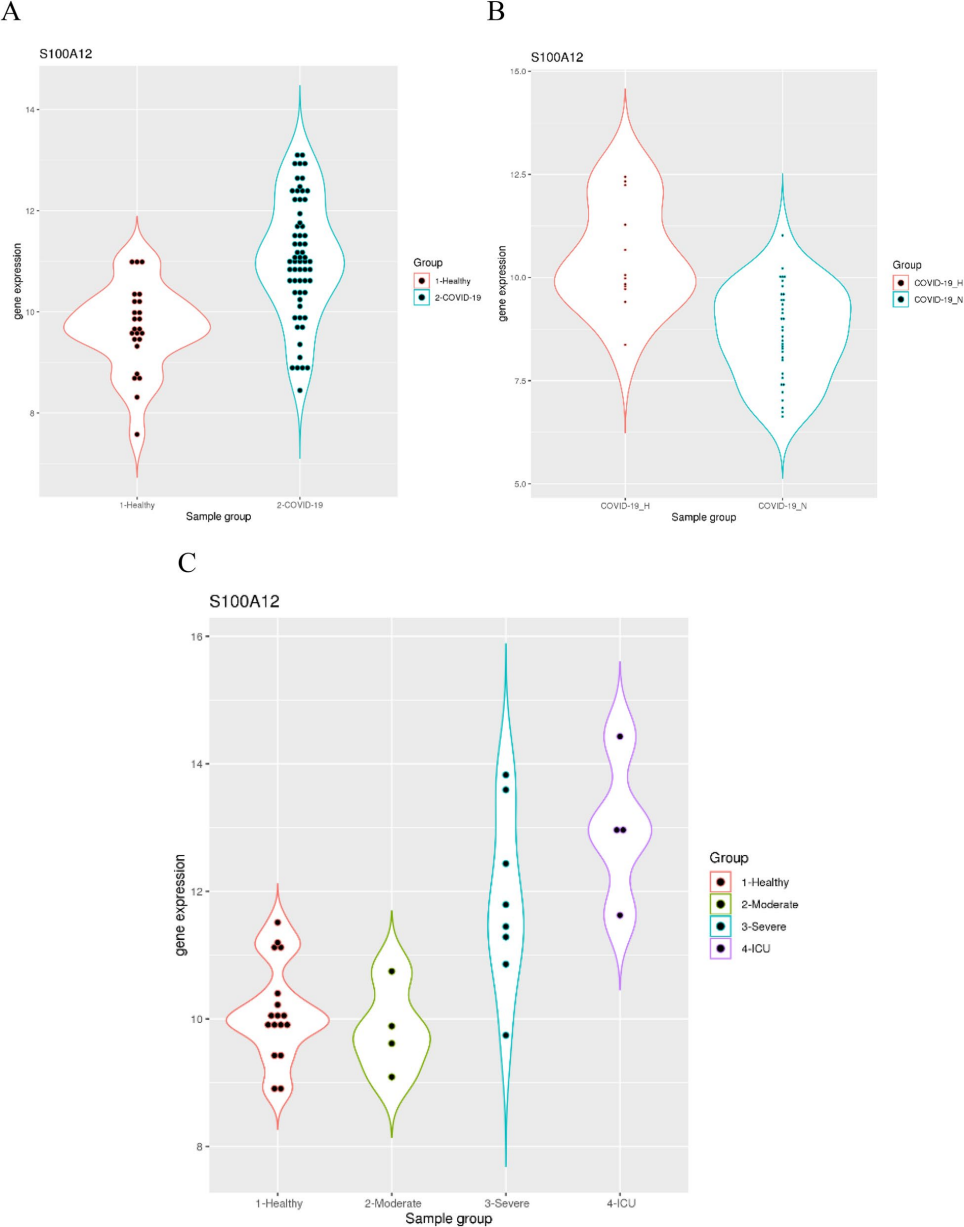
S100A12 expression elevated in severe and critical COVID-19 patients. (**A**) upper left, comparison of *S100A12* expression in healthy controls and COVID-19 patients. (**B**) upper right, comparison of *S100A12* expression in hospitalized and non-hospitalized COVID-19 patients. (**C**) lower panel, comparison of *S100A12* expression in healthy controls and patients with COVID-19 at three severity levels (moderate, severe and ICU).

The more direct question is whether *S100A12* expression is elevated in patients with severe COVID-19 infection. The dataset GSE152641 included samples from healthy controls and hospitalized COVID-19 patients (Fig. 4A). It was evident that *S100A12* expression was significantly elevated in the COVID-19 infection group compared to the healthy control group (*p* = 1.02e-7). More specifically, 31 of the 62 hospitalized COVID-19 patients (50%) had *S100A12* expression above the highest value in the control group. Another dataset GSE161731 included samples from both hospitalized and non-hospitalized COVID-19 patients (Fig. 4B). It was evident that *S100A12* expression was significantly elevated in the hospitalized COVID-19 group compared to the non-hospitalized COVID-19 group (*p* = 0.00039). Thus, *S100A12* expression is elevated in a subgroup of hospitalized COVID-19 patients.

Another dataset GSE152418 included samples from healthy controls and COVID-19 patients with various severity (Fig. 4C). The *S100A12* expression was not significantly different between the moderate COVID-19 infection group and the healthy control group (*p* = 0.51). However, *S100A12* expression was significantly elevated in the severe COVID-19 infection group (*p* = 0.0066) and ICU group (*p* = 0.0054) compared to the moderate COVID-19 infection group. Similar results were found in another study with similar experimental design^57^. In that study, *S100A12* expression was not significantly different between the moderate cases and the healthy controls, but it was significantly elevated in the severe cases compared to the moderate cases (log2FC = 3.58, q = 0.00034). Therefore, *S100A12* expression is indeed elevated in patients with severe COVID-19 infection.

### *S100A12* expression is correlated with future disease severity indexes in COVID-19 infection

**Figure 5:**
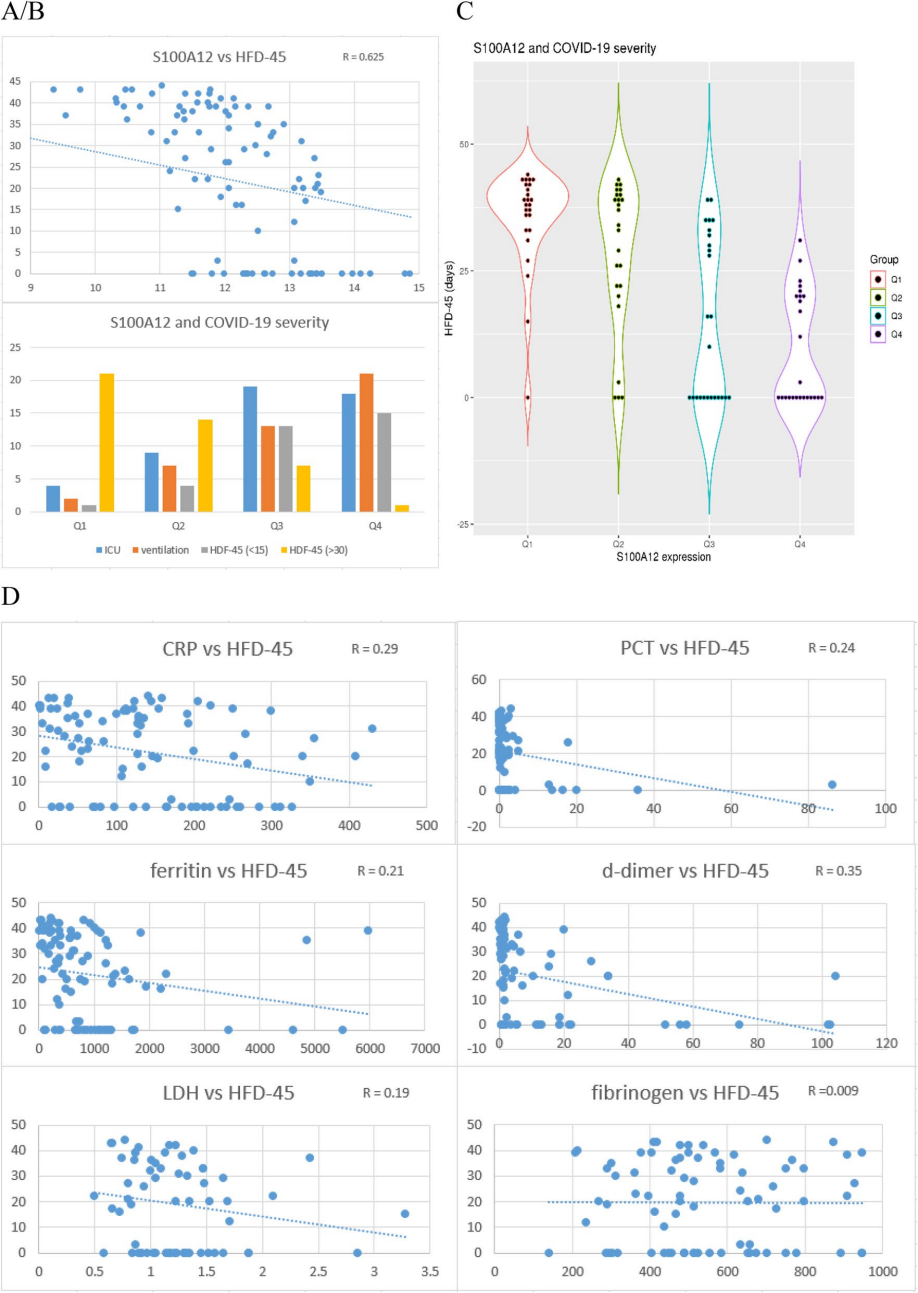
S100A12 expression highly correlated with future disease severity in COVID-19 patients. (**A**) upper left, scatter plot, correlation of *S100A12* expression with severity index HFD-45 in COVID-19 patients. (**B**) upper left, histogram plot, comparison of severity indexes in four groups of COVID-19 patients based on the *S100A12* expression. The severity indexes include the status of admission to ICU, use of mechanical ventilation, and small or large HFD-45 values. (**C**) upper right, distribution of HFD-45 scores in four groups of COVID-19 patients based on the *S100A12* expression. (**D**) lower panel, correlation of established markers with HDF-45.

To go one step further, it will be interesting to find out whether *S100A12* expression at hospital admission is correlated with future quantitative indexes of disease severity in COVID-19 patients (assessed retrospectively). The dataset GSE157103 included samples from COVID-19 patients with various severity using hospital free days at day 45 of hospital admission (HFD-45) as a disease severity index (Fig. 5A). It was evident that *S100A12* expression was robustly correlated with disease severity as measured by HFD-45 (r = 0.625), which was much better than the established prognostic markers such as CRP, PCT, ferritin, d-dimers, LDH and fibrinogen (r between 0.009 and 0.35, Fig. 5D). In contrast, the expression of ISGs displayed extremely weak correlation with HFD-45 (r = 0.025 for *IFI27* and r = 0.11 for *RSAD2*).

For more detailed examination, the samples were equally divided into four groups based on the *S100A12* expression (25 patients in each group). Using the Q1 group as the reference, the Q2 group was only marginally different from the Q1 group in terms of HFD-45 (*p* = 0.062), while the other two groups were significantly different from the Q1 group (*p* = 4.25e-6 for the Q3 group, and *p* = 2.23e-11 for the Q4 group) (Fig. 5C). More specifically, the Q1 group had only one patient with HFD-45 less than 15 days, while it was 4, 13 and 15 patients in the Q2, Q3, and Q4 groups, respectively (Fig. 5B). This can be considered as having fourfold, 13-fold and 15-fold increased risk of having severe patients in the Q2, Q3, and Q4 groups, respectively. On the other hand, the Q1 group had 21 patients with HFD-45 more than 30 days, while it was 14, 7 and 1 patients in the Q2, Q3, and Q4 groups, respectively (Fig. 5B).

As additional assessment of disease severity, information on ICU admission and mechanical ventilation were included in this study. There were 4 patients admitted to ICU in the Q1 group, while it was 9, 19 and 18 patients in the Q2, Q3 and Q4 groups, respectively (Fig. 5B). In addition, there were only 2 patients using mechanical ventilation in the Q1 group, while it was 7, 13 and 21 patients in the Q2, Q3 and Q4 groups, respectively.

The same study included 26 non-COVID-19 patients. Due to the limited sample size, these patients were only divided into two groups based on the *S100A12* expression (H1 and H2 groups). The H1 group had 4 of the 13 patients admitted to ICU, while the H2 group had 12 of the 13 patients admitted to ICU. None of the patients in the H1 group used mechanical ventilation, while 9 of the 13 patients in the H2 group used mechanical ventilation. Thus, *S100A12* expression seems to be a more general indicator of disease severity, which deserves further investigation.

## Discussion

A variety of biomarkers have been proposed for the prognosis of disease severity in COVID-19 patients, and some of the more accessible ones have been validated in many independent studies. However, all of the reported biomarkers only have modest prognostic power. In the current study, it has been demonstrated that *S100A12* expression in the whole blood is a robust marker for severe infection and severe respiratory viral infection. Not surprisingly, it is also a marker for severe COVID-19 and is robustly correlated with future quantitative indexes of COVID-19 severity, much better than the established prognostic biomarkers. Due to limited data availability specifically for COVID-19, relevant data on severe infection and severe respiratory viral infection has been extensively investigated in this study. The intention of this study is to find a biomarker universally applicable to infection in general and more specifically to respiratory viral infection. If the purpose is find biomarkers unique to COVID-19, a lot more data on COVID-19 will be required for the conclusion to be convincing.

I shall further clarify that the aim of this work is to find novel prognostic markers for COVID-19, not diagnostic markers. The field of COVID-19 diagnosis is quite mature. There are dozens of commercial kits available with high sensitivity and specificity. There are also commercial kits available with fast and convenient detection of COVID-19. Additionally, there are commercial kits for simultaneous detection of dozens of common pathogens including COVID-19. As for host response to different types of infection, people have also derived gene panels to differentiate common infection types^53,54^. However, it may not be practical to clinical use because hundreds of genes are required.

The reason for the selection of *S100A12* expression in this study is that it stands out to be the most prominent marker for bacterial infection in our previous works. Since most genes including immune-related genes are multi-functional, it is conceivable that the function of *S100A12* is not limited to the response to bacterial infection. It has been well-established that the signature of host response to viral infection is ISGs. However, the severity of COVID-19 does not seem to be correlated with the expression of ISGs at all. It is possible that patients with severe COVID-19 may activate pathways involving *S100A12* in addition to the initial activation of interferon signaling pathways as a response to the overwhelming infection. The exact role of *S100A12* in the response to severe infection will require more in-depth investigation.

It shall be noted that ISGs as the signature for host response to respiratory viral infection was mainly derived from mild infections or even human challenge experiments. Host response to severe respiratory viral infection including COVID-19 could be much more complicated. It’s not entirely surprising that “bacterial signature” such as *S100A12* is activated in severe respiratory viral infection. It’s possible that sometimes viral infection is simply overwhelming for the immune systems of certain infected individuals and interferon response by itself is way too weak to stop virus replication and systemic damage to the human host. More specifically, neutrophils which express high level of *S100A12* could be heavily involved in host response to severe COVID-19 even at the early stage of the disease development, including both neutrophil expansion and neutrophil-related gene activation.

## Data Availability

All relevant data are publicly available as described in the manuscript.
